# On-Demand Isolation of Bacteriophages Against Drug-Resistant Bacteria for Personalized Phage Therapy

**DOI:** 10.3389/fmicb.2015.01271

**Published:** 2015-11-13

**Authors:** Sari Mattila, Pilvi Ruotsalainen, Matti Jalasvuori

**Affiliations:** Department of Biological and Environmental Science, Centre of Excellence in Biological Interactions, University of JyväskyläJyväskylä Finland

**Keywords:** antibiotic resistance, ESBL, MRSA, phage therapy, phage cocktails, bacteriophages

## Abstract

Bacteriophages are bacterial viruses, capable of killing even multi-drug resistant bacterial cells. For this reason, therapeutic use of phages is considered as a possible alternative to conventional antibiotics. However, phages are very host specific in comparison to wide-spectrum antibiotics and thus preparation of phage-cocktails beforehand against pathogens can be difficult. In this study, we evaluate whether it may be possible to isolate phages on-demand from environmental reservoir. We attempted to enrich infectious bacteriophages from sewage against nosocomial drug-resistant bacterial strains of different medically important species in order to evaluate the probability of discovering novel therapeutic phages. Stability and host-range were determined for the acquired phages. Our results suggest that on-demand isolation of phages is possible against *Pseudomonas aeruginosa, Salmonella* and extended spectrum beta-lactamase *Escherichia coli* and *Klebsiella pneumoniae*. The probability of finding suitable phages was less than 40% against vancomycin resistant *Enterococcus* and *Acinetobacter baumannii* strains. Furthermore, isolation of new phages against methicillin resistant *Staphylococcus aureus* strains was found to be very difficult.

## Introduction

Antibiotic resistance is an emerging global health crisis, resulting from the continuous use (and misuse) of antibiotics in healthcare, farming industry, and elsewhere (Cantas et al., 2013; World Health Organization [WHO], 2014). Phage therapy refers to the utilization of bacteriophages (or just phages, viruses infecting bacteria) to treat bacterial diseases (Abedon et al., 2011). Given the increasing number of drug-resistant bacterial infections, especially within hospital settings, the exploration of alternatives to conventional antibiotics has become an important research objective (Finch, 2011; Sommer and Dantas, 2011). Bacteriophages are very abundant (Hendrix et al., 1999) and every bacterium is likely to have their own specific viruses that could be utilized as antibacterial agents (Clokie et al., 2011; Flores et al., 2011; Örmälä and Jalasvuori, 2013). Historically, phages were used therapeutically already in the early 20th century (Sulakvelidze et al., 2001). Yet, the discovery of broadly effective antibiotics led to the demise of the development of phage therapy in western countries and only as the antibiotics are starting to fail there has been a serious attempt to restore the old tool. However, the second coming of phage therapy faces challenges regarding to the strict regulatory guidelines and the development of effective therapeutic practices (Gill and Hyman, 2010; Lu and Koeris, 2011; Keen, 2012). Yet, phage therapy can provide an evolutionarily sustainable alternative to conventional antibiotics, should we be able to adjust our regulations and procedures to meet the special requirements of phage based medicine (Keen, 2012; Örmälä and Jalasvuori, 2013).

It is important to note that phages infect bacterial hosts very selectively. Often, the narrow host-range is considered as an advantage over traditional antibiotics since phage treatment can focus accurately on the pathogen without harming commensal bacterial flora (Loc-Carrillo and Abedon, 2011). On the other hand, bacteria develop resistance also to phages rapidly, and thus the achieved antibacterial effect may be transient (Hyman and Abedon, 2010; Labrie et al., 2010). When multiple different phages are used simultaneously in a phage cocktail, development of resistance is less likely (Skurnik et al., 2007; Chan et al., 2013). However, it is challenging to obtain a set of phages that is effective against all variants of a given pathogen (Pirnay et al., 2011; Chan et al., 2013). There can be a tradeoff between the host range and the therapeutic efficacy of a cocktail for a specific species of bacteria: when the number of phages in a cocktail increases in an effort to increase the host range of the cocktail, the number of phages against a specific strain of bacteria may decrease. Therefore, the host specificity of phages, while in theory beneficial, poses a practical problem when combined with the rapidly emerging resistant phenotypes.

In principle, it is possible to acquire bacteriophages on-demand to treat, for example, infections that are resistant to all known antibiotics and off-the-shelf (standardized) phage-therapy products (Keen, 2012; Örmälä and Jalasvuori, 2013). Tailoring a therapeutic cocktail personally for each patient would allow the cocktails to comprise phages that are effective against the bacterial strains responsible of the infection (Pirnay et al., 2011; Chan et al., 2013). Therefore and in comparison to premade cocktails, a personalized phage therapy does not carry a surplus of ineffective phages. Indeed, there are older studies suggesting that tailored phage treatments are several times more effective compared to standardized cocktails (Zhukov-Verezhnikov et al., 1978), and thus effective phage-therapy practices to treat constantly changing bacterial pathogens may depend on the adjustment of the treatment to the causative agent (Keen, 2012).

Generating a personal set of phages requires that the pathogen is isolated and, then, effective bacteriophages obtained against it. One possible way for identifying suitable viruses is to have a variety of bacteriophages isolated and prepared beforehand and then the causative pathogen screened through the phage-library (Chan et al., 2013). Alternatively, phages may be isolated as needed from environmental reservoirs. In some cases, the latter option may be inevitable due to the lack of infectious phages in the premade libraries against all possible bacterial variants. Ultimately, environment serves as the only source of practically endless phage variety and thus exploitation of the environmental resources forms the basis for personalized phage medicine.

While phages are known to be abundant, it is obvious that all environments cannot contain infective phages against all different bacterial hosts (see e.g., Flores et al., 2011; Atanasova et al., 2012). To the best of our knowledge, the probability of finding therapeutically useful phages against different resistant pathogens on-demand has not been studied *per se* despite the fact that it is likely to be the limiting factor in attempts to update premade cocktails or to generate on-demand personalized therapies (Chan et al., 2013). As an example, hospital acquired wound infections have been suggested to be especially suitable target for phage therapy as the causative agents are generally resistant to various antibiotics (Loc-Carrillo et al., 2012). Yet, there might be multiple different bacterial species present in these infections, including, e.g., *Staphylococcus aureus*, *Enterococcus faecium*, *Escherichia coli*, *Klebsiella pneumoniae*, *Pseudomonas aeruginosa*, and *Acinetobacter baumannii* (Agnihotri et al., 2004). Therefore, a successful phage-based treatment can be dependent on the practicality of being able to simultaneously and rapidly isolate new durable phages against very different pathogens.

In this study, we provide an evaluation of the on-demand isolation of phages against the most common hospital borne resistant pathogens: methicillin resistant *S. aureus* (MRSA), extended spectrum beta-lactamase (ESBL) *E. coli* and *K. pneumoniae*, multi-drug resistant (MDR) *P. aeruginosa*, vancomycin resistant *Enterococcus* (VRE), *A. baumannii* and different *Salmonella* species. All aforementioned species are also listed in CDC’s report on the top 18 drug-resistant threats to the United States in 2013 (CDC, 2013). These bacteria commonly cause infections of skin, lung and urinary tract, as well as foodborne infections among others and affect people all around the world disregarding their background (CDC/FDA/NIH, 2011).

Sewage is known to be an optimal resource of phages (Lobocka et al., 2014), thus a wastewater treatment plant in Jyväskylä, Finland (Nenäinniemi) was used as the environmental reservoir for phage hunt. The stability of the acquired viruses and their cross-infectivity on other potential host strains were determined.

We demonstrate vast differences in probabilities of finding novel phages against different hosts by using enrichment method for isolation. There appears to be severe constraints in isolating phages on-demand against pathogens like MRSA. On the other hand, it seems feasible to obtain phages against ESBL positive *E. coli* and *K. pneumoniae* as well as *P. aeruginosa*.

## Materials and Methods

### Bacteria Strains and Culturing Conditions

Bacterial strains used in this study were mostly purchased from Medix Laboratories or acquired from Turku University Hospital (**Supplementary Table S1**). One *Klebsiella* strain and four *Enterococccus* strains were obtained from commercial culture collections. Aside from six bacterial strains, all had caused (antibiotic resistant) human infections and thus they represent pathogens that could have been treated with phages. Overall, we obtained 12 MRSA strains, 16 *E. coli* ESBL strains, 6 *K. pneumonia* (ESBL) strains, 17 *P. aeruginosa* MDR strains, 9 *A. baumannii* strains, 10 *E. faecium* (VRE) strains, 4 *Enterococcus faecalis* (VRE) strains, and 9 different *Salmonella* strains. Detailed characterization of the bacterial strains was beyond the scope of this paper.

All bacteria were cultured in Lysogeny Broth (LB) -medium (Sambrook et al., 1989) at +37°C shaken 230 rpm (*Enterococcus* strains were cultivated without shaking).

### Isolation Protocol

The following isolation protocol with slight modification in individual experiments was used throughout the study. Either unprocessed sewage samples or supernatants of turbid samples (centrifuged 3000–6000 *g* in Megafuge 1.0R, Heraeus, or in Eppendorf centrifuge 5702 R, 10–15 min at +4°C) were used in the enrichment steps. In cases where previous isolation attempts had failed to yield phages, the supernatant was also filtrated through a 0.45 μm filter to remove all remaining bacterial cells. The first enrichment step was conducted using 20–30 ml of sewage water filled up to 30–40 ml with LB-broth, depending on the volume of collected sewage samples. The target bacterial strain was added (50–200 μl o/n culture grown in LB-broth, 300 μl in case of *E. faecium* and *E. faecalis*) to enrich (potential) phages in the sample. These enrichments were cultivated overnight at +37°C, shaken 230 rpm. Bacteria from this enrichment culture were removed by centrifugation (3000–6000 *g* in Megafuge 1.0R, Heraeus, or in Eppendorf centrifuge 5702 R, 15–20 min, +4°C) and filtration (0.2 or 0.45 μm filter). The amount of potential phages in a 2.5 ml sample of the bacteria-free enrichment were further amplified by adding 2.5 ml of LB-broth and 50–100 μl of the target host bacterium and were grown overnight as above. The sample from this second enrichment step was centrifuged at 13 000 *g* for 15 min at room temperature and at least 10 μl the supernatant was plated on a LB-agar containing petri dish along with 100–300 μl of the host strain and 3 ml of melted 0.7% soft-agar. The plates were incubated overnight at +37°C. If plaques were observed on the bacterial lawn, a separate plaque was picked and transferred into 500 μl of LB-broth. A sample from this plaque-stock was further plated on the same host strain. Plaque-purification was performed three times for all discovered phages in order to isolate a single homogenous phage from the potentially heterogeneous phage mix that may have been present in the initial enrichment.

Due to poor isolation success for *S. aureus*, different modifications of the above-described method were used for enriching phages. The volume of the first enrichment step as well as the number of enrichment steps was increased (120 ml sewage sample + 70 ml L broth + 1 ml host overnight cultures in the first step). Rotation speed during shaken cultivation steps was varied between 100, 120, 180, or 360 rpm. In addition, samples from different sources were used for phage enrichment (River in Ljubljana, Slovenia, a water-lock sample from the Helsinki university hospital and soil samples from a livestock farm). These samples were not included in analysis of isolation success from sewage.

### Preparation of Phage Stock

Semi-confluent plates (i.e., plates of which about half of the area is covered by phage induced plaques and the rest is bacterial lawn) were prepared by plating 100 μl of host strain (300 μl of *Enterococcus* strains) and 3 ml of melted soft-agar with appropriate dilution of the phage stock. Plates were incubated overnight at +37°C. The soft-agar layers of semi-confluent plates were combined with 2.5–5 ml of LB-broth/plate. The combination was incubated for 4 h at +37°C, 230 rpm, and centrifuged at 6000 *g* for 15 min at +4°C (Megafuge 1.0R, Heraeus). If we were unable to get semi-confluent plates, we used as a combination “over-infected” plates supplied with 100–700 μl of the overnight-cultivated host strain. The supernatant was filtered (0.2 μm filter) and stored at +4°C.

### Cross Infection Tests

All phages were used to cross-infect all different bacterial strains of its original host species (excluding *P. aeruginosa* phages as only half of them were used) for preliminary evaluation of their host range. Cross-infection tests were done by spotting 8 μl of phage stock dilution (1:10 or 1:100) on 100 μl bacterial overnight culture in soft-agar (0.7%). Plates were incubated at +37°C overnight. Formation of less opaque spots on the bacterial lawn was scored as a successful infection.

### Phage Stock Stability

The titer of each phage stock was determined by standard double agar overlay method by plating a dilution series (10^-2^–10^-8^) immediately after preparation of the stock. Titer of the stock was determined again after 1-month storage (+4°C) to estimate the stability of the stock in LB-medium.

## Results

We evaluated the feasibility for generating a personalized phage-product on-demand against different bacterial pathogens. We chose bacterial species from seven different genuses that are responsible for the majority of hospital acquired bacterial infections, namely *Escherichia*, *Salmonella*, *Klebsiella*, *Pseudomonas*, *Staphylococcus*, *Enterococcus*, and *Acinetobacer*. Total of 283 phage isolation attempts were conducted for 83 different host strains. Overall 108 bacteriophages were discovered. All of these viruses were characterized for their plaque morphology and stability (described individually for each virus in **Supplementary Table S2**).

**FIGURE 1:**
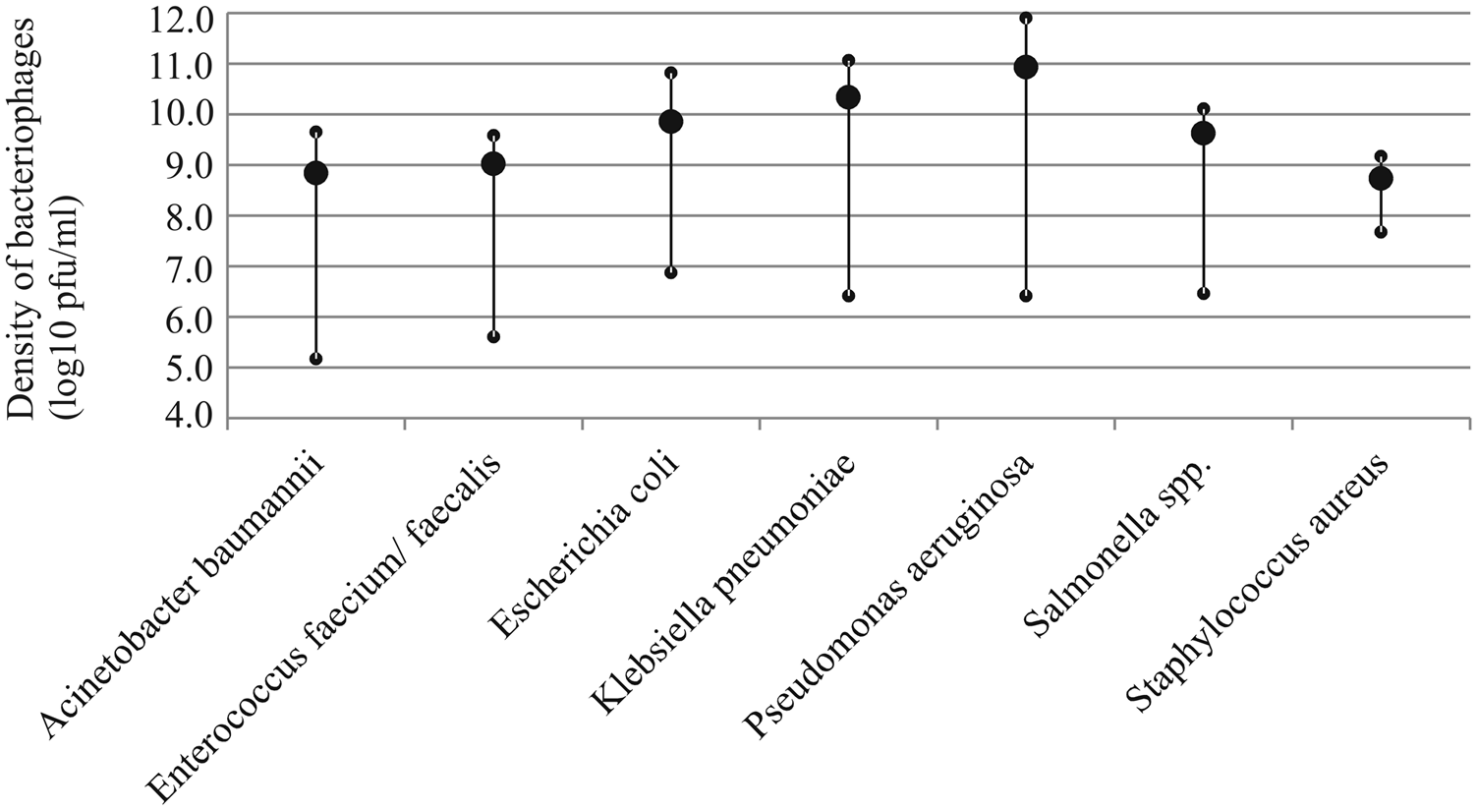
**Average density of infectious bacteriophage particles in the prepared stocks of each host species (large circle).**Small circles indicate the maximum and minimum values observed.

Phages were isolated via three consecutive plaque-picking steps to avoid mixed-culture stocks. Due to different plaque morphologies and titers, the preparation of phage stocks was adjusted for each phage. However, no actual optimization of phage production was carried out. The density of viable phage particles was measured immediately after the preparation of the stock (**Figure 1**). In order to determine their viability for acute use, the number of viable particles was re-measured 1 month later (see summary in **Table 1**). On average, the titers of the stocks decreased around 0.5 log10 during the 1-month storage in L-broth in 4°C. However, for some phages of *Enterococcus*, the titers could no longer be resolved. Phage-specific titers and plaque morphologies are listed in **Supplementary Table S2**.

**Table 1:** Summary of the decrease in phage titers as observed after 1-month storage at +4°C.

Host bacterium	Average decrease in titer (log10)
*Acinetobacter baumannii*	0.973
*Enterococcus faecium/ faecalis*	0.222
*Escherichia coli*	0.496
*Klebsiella pneumoniae*	0.594
*Pseudomonas aeruginosa*	0.437
*Salmonella* sp.	0.529
*Staphylococcus aureus*	0.491


The probability for finding an infectious bacteriophage from sewage for different host bacterium varied substantially (**Table 2**). Namely, phages for only a single *S. aureus* strain, SA10, were discovered in total of 117 enrichment attempts (the phages specific to the one *S. aureus* strain were obtained at the same time and they produced visually identical plaques, thus we selected only one of these phages for subsequent analyses). Conversely, almost every isolation attempt yielded a bacteriophage for *E. coli*, *K. pneumoniae*, *P. aeruginosa*, and *Salmonella* strains. Phage isolation for *Acinetobacter* and *Enterococcus* had success rates between 30 and 40%. Given the medical importance of MRSA, we decided to investigate whether alternative source materials would be more suitable for discovering phages. We obtained water samples from a water lock situated in a room used to treat MRSA-patients in Helsinki University Hospital. Two phages for a single strain (SA10) were found from these samples. A single MRSA-specific bacteriophage was isolated from a set of soil samples acquired from a livestock farm. Also, a water sample from river Ljubljana, Slovenia, produced a single bacteriophage for strain SA10. Yet, we failed to find a single phage for any of the ten other MRSA-strains used in the isolation attempts.

**Table 2:** Probability for discovering a bacteriophage from a sewage sample against different pathogens.

Bacterial pathogen	Mean hit %^∗^	Isolation attempts	Number of strains hit
*Acinetobacter baumannii*	38.9	34	5/9
*Enterococcus faecium/faecalis*	33.9	27	5/14
*Escherichia coli*	90.6	35	15/16
*Klebsiella pneumoniae*	83.3	15	6/6
*Pseudomonas aeruginosa*	79.4	44	15/17
*Salmonella* sp.	88.9	11	8/9
*Staphylococcus aureus*	6.1	117	1/12


*^∗^As calculated over the bacterial strains of the given species.*

**FIGURE 2:**
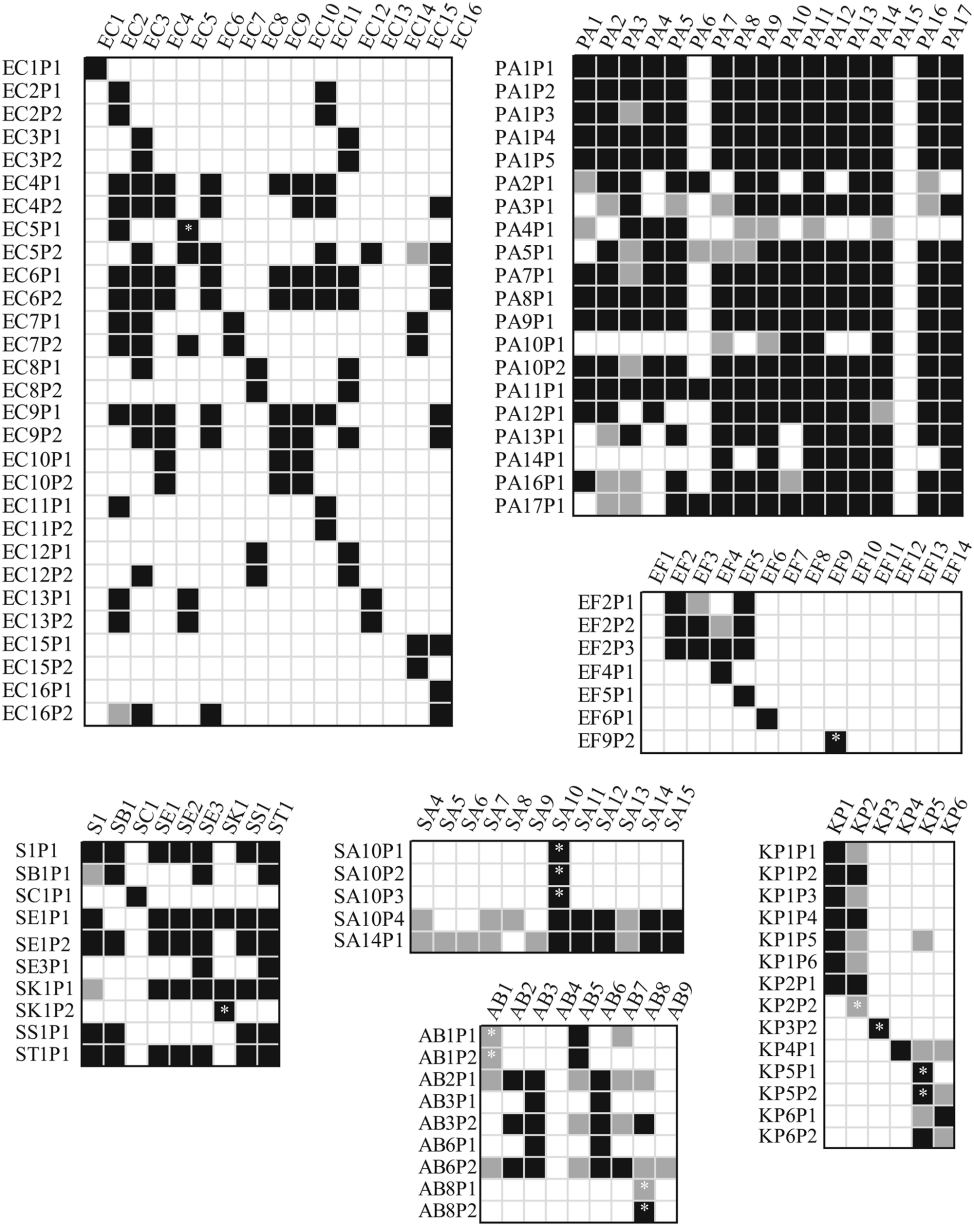
**Cross-infectivity of the isolated bacteriophages.**Measurements where less than 10^5^ pfu/ml were used are indicated with an asterisk. Only half of the isolated *Pseudomonas* phages were used in the experiments. White background indicates no lysis area, black marks clearly detected lysis area and light gray indicates very dim lysis area in the spot test. AB, *Acinetobacter baumannii*; EC, *Escherichia coli*; EF, *Enterococcus faecium* or *faecalis*; KB, *Klebsiella pneumoniae*; PA, *Pseudomonas aeruginosa*; S, *Salmonella* sp., and SA, *Staphylococcus aureus*.

As presented in **Figure 2**, we studied the host-range of the obtained phages in order to determine their cross-infectivity and thus the potential to combine previously isolated phages into phage-cocktails. Aside from a couple of exceptions, almost all phages isolated for any given *P. aeruginosa* strain could also infect majority of the other strains. However, we neither found any phages for strain PA15 nor did any of the other phages infect this strain. In addition, only 4 out of 20 tested *Pseudomonas* phages infected strain PA6. Detailed characterization of these particular strains was beyond the scope of this paper.

Along with *Pseudomonas* phages, some of the *Salmonella* phages had a wide host range. *E. coli* phages tended to infect more than one strain, except EC1P1, EC11P2, EC15P2, and EC16P1. For other bacterial species, isolated phages generally had less alternative hosts, if any, indicating that a rapid preparation of a personalized phage-cocktail is likely to require multiple separate but simultaneous phage enrichments using a single bacterial strain as a host. Especially, *Klebsiella* and *Enterococcus* phages are very host specific. Sometimes phage stocks produced only a dim inhibition area on alternative hosts (presented as light gray coloring in **Figure 2**). This suggests that something, but not necessarily the phage in the prepared stocks was restricting the growth of the bacterium. Furthermore, phages isolated for any particular host often had similar infection patterns. This suggests that additional isolation attempts using the same isolation source for enrichment may not be the best choice for improving the host-range of the cocktail.

## Discussion

Due to the enormous variety of bacteriophages in environmental reservoirs, on-demand isolation of novel phage-antibacterials is a potential way to generate a personalized medicine for treating bacterial infections that are resistant to conventional drugs. In this study, we evaluated the feasibility of isolating phages for such therapeutic cocktails.

The efforts required to find phages differs substantially between bacterial species. Phages can be readily discovered for *E. coli*, *K. pneumoniae*, *P. aeruginosa*, and *Salmonella* species. Although virus production was neither optimized nor standardized in this study, phages of these hosts also readily generated high-density virus stocks (**Figure 1**). In contrast, we found it very challenging to isolate phages against *Staphylococcus* strains despite of several attempts that were conducted at different times of year and from multiple sources (sewage, river, hospital water lock, and livestock farm soil samples). It was also more laborious to isolate phages for *E. faecium* and *faecalis* and *A. baumannii*, although it must be noted that we had only handful of these strains and we performed only few isolation attempts for them. Nevertheless, based on the results, the on-demand discovery of phages appears to be feasible for some but not all bacteria. This highlights the importance of premade wide-host range cocktails or the existence of other antimicrobial solutions against species such as *S. aureus* (such as the one developed by Kelly et al., 2011). Also, teixobactin, the first new potential antibiotic to be discovered in 30 years is very effective against bacteria lacking the outer membrane (such as *S. aureus* and *Enterococcus*; Ling et al., 2015). Yet, gram-negative pathogens with the impermeable outer membrane (e.g., *E. coli*, *Salmonella*, *K. pneumonia*, and *P. aeruginosa*) are inherently resistant to antibiotics like teixobactin, but contrastingly appear to be suitable targets for obtaining a cocktail from environmental reservoir (sewage) as needed. Also, better preservability and wider host-range of these phages supports the on-demand isolation approach. While conventional tools for antibiotic development may still remain relevant, in the face of worsening world-wide antibiotic resistance crisis we should be actively exploring these promising alternatives in order to retain the upper hand against all pathogens.

Generalization of the obtained results must be done while acknowledging the potential sources of error. First, while we collected our sewage samples at different times (over the timespan of almost 2 years), only a single wastewater management plant was used. Although the biological material in these plants changes constantly, the phage populations may still be substantially different in different plants, thus possibly skewing the chances for finding phages against certain species. Moreover, the host ranges of some phages appear identical, suggesting that the hosts themselves may be genetically very close to one another. Second, albeit we performed several hundred isolation attempts, just a few isolations were performed for any particular strain and thus the achieved probabilities should be treated as a case study rather than an exhaustive evaluation. Third, we did not perform an in-detail characterization for the isolated phages. Such characterization, at least to some extent, will be necessary during actual therapy practices (Skurnik et al., 2007; Merabishvili et al., 2009; Keen, 2012), as bacteriophages are known to carry undesirable genes coding for toxins and antibiotic resistances (Loc-Carrillo and Abedon, 2011). However, separating lytic phages from temperate phages (possibly when accompanied with genome sequencing and analysis) should be enough and feasible for the rapid assessment of safety (Chan et al., 2013). Also, phage stocks have to be purified from (host-bacterium generated) endotoxins before therapeutic use (Keen, 2012). These steps were not performed or their effects on phages evaluated in this study.

## Conclusion

The success of on-demand isolation of phages appears to be critically dependent on the bacterial host. Promisingly, against pathogens for which conventional antibiotics are becoming the least useful, such as ESBL *E. coli* and *K. pneumoniae*, personalized phage therapy could be considered as a potential alternative.

## Conflict of Interest Statement

The authors declare that the research was conducted in the absence of any commercial or financial relationships that could be construed as a potential conflict of interest.
