# Supplementary material for: On-Demand Isolation of Bacteriophages Against Drug-Resistant Bacteria for Personalized Phage Therapy
Source: Front Microbiol. 2015 Nov 13;6:1271. doi: 10.3389/fmicb.2015.01271 (PMC4643220; doi:10.3389/fmicb.2015.01271)
Supplement: Supplementary file 1 [file Table_1.PDF]

Table S1. Bacterial strains used in this study.

Bacterial strains	Short name	Source
Acinetobacter baumannii 57163	AB1	Turku University Hospital
Acinetobacter baumannii 59999	AB2	Turku University Hospital
Acinetobacter baumannii 62060	AB3	Turku University Hospital
Acinetobacter baumannii 20692	AB4	Turku University Hospital
Acinetobacter baumannii 57163	AB5	Turku University Hospital
Acinetobacter baumannii 18243	AB6	Turku University Hospital
Acinetobacter baumannii 26006	AB7	Turku University Hospital
Acinetobacter baumannii 18510	AB8	Turku University Hospital
Acinetobacter species 18560	AB9	Turku University Hospital
Enterococcus faecium 61285	EF1	Turku University Hospital
Enterococcus faecalis 58897	EF2	Turku University Hospital
Enterococcus faecalis	EF3	ATCC 19433
Enterococcus faecalis	EF4	ATCC 29212
Enterococcus faecalis	EF5	ATCC 33186
Enterococcus faecium	EF6	ATCC 9790
Enterococcus faecium 59776	EF7	Turku University Hospital
Enterococcus faecium 61244	EF8	Turku University Hospital
Enterococcus faecium 61027	EF9	Turku University Hospital
Enterococcus faecium 60803	EF10	Turku University Hospital
Enterococcus faecium 60734	EF11	Turku University Hospital
Enterococcus faecium 60703	EF12	Turku University Hospital
Enterococcus faecium 60457	EF13	Turku University Hospital
Enterococcus faecium 60145	EF14	Turku University Hospital
Escherichia coli 10AE5909	EC1	Medix
Escherichia coli 12UM05186	EC2	Medix
Escherichia coli 10UU11258	EC3	Medix
Escherichia coli 11UT10019	EC4	Medix
Escherichia coli 11AN03027	EC5	Medix
Escherichia coli 11UT12639	EC6	Medix
Escherichia coli 11UO03492	EC7	Medix
Escherichia coli 11UU07697	EC8	Medix
Escherichia coli 11UM05271	EC9	Medix
Escherichia coli 57262	EC10	Turku University Hospital
Escherichia coli 57294	EC11	Turku University Hospital
Escherichia coli 57189	EC12	Turku University Hospital
Escherichia coli 57253	EC13	Turku University Hospital
Escherichia coli 55027	EC14	Turku University Hospital
Escherichia coli 56895	EC15	Turku University Hospital
Escherichia coli 57361	EC16	Turku University Hospital
Klebsiella pneumoniae	KP1	DSM681
Klebsiella pneumoniae 10UO03898	KP2	Medix
Klebsiella pneumoniae 61705	KP3	Turku University Hospital
Klebsiella pneumoniae 61784	KP4	Turku University Hospital
Klebsiella pneumoniae 61837	KP5	Turku University Hospital
Klebsiella pneumoniae 61794	KP6	Turku University Hospital
Pseudomonas aeruginosa 61841	PA1	Turku University Hospital
Pseudomonas aeruginosa 61823	PA2	Turku University Hospital
Pseudomonas aeruginosa 61790	PA3	Turku University Hospital
Pseudomonas aeruginosa 61432	PA4	Turku University Hospital
Pseudomonas aeruginosa 11AN03663	PA5	Medix
Pseudomonas aeruginosa 26153	PA6	Turku University Hospital
Pseudomonas aeruginosa 62314	PA7	Turku University Hospital
Pseudomonas aeruginosa 62263	PA8	Turku University Hospital
Pseudomonas aeruginosa 62224	PA9	Turku University Hospital
Pseudomonas aeruginosa 62206	PA10	Turku University Hospital
Pseudomonas aeruginosa 62180	PA11	Turku University Hospital
Pseudomonas aeruginosa 62181	PA12	Turku University Hospital
Pseudomonas aeruginosa 62172	PA13	Turku University Hospital
Pseudomonas aeruginosa 62109	PA14	Turku University Hospital
Pseudomonas aeruginosa 62090	PA15	Turku University Hospital
Pseudomonas aeruginosa 62069	PA16	Turku University Hospital
Pseudomonas aeruginosa 61932	PA17	Turku University Hospital
Salmonella, Group B, ESBL strain 12FB01687	SB1	Turku University Hospital
Salmonella, Group C, 13FB10784	SC1	Turku University Hospital
Salmonella enteritis (36.)	SE1	Turku University Hospital
Salmonella enteritidis 13FB9205	SE2	Turku University Hospital
Salmonella enteritidis FB11214	SE3	Turku University Hospital
Salmonella Kreber (37.)	SK1	Turku University Hospital
Salmonella stanley FB3820	SS1	Turku University Hospital
Salmonella typhimurium FB7595	ST1	Turku University Hospital
Salmonella, quality control strain 18048	S1	Turku University Hospital
Staphylococcus aureus 11AN02972	SA4	Medix
Staphylococcus aureus 10MR01905	SA5	Medix
Staphylococcus aureus 11AE06590	SA6	Medix
Staphylococcus aureus 11AE07165	SA7	Medix
Staphylococcus aureus 11AN03312	SA8	Medix
Staphylococcus aureus 10AN02929	SA9	Medix
Staphylococcus aureus 10AE05905	SA10	Medix
Staphylococcus aureus 10AE06192	SA11	Medix
Staphylococcus aureus 60820	SA12	Turku University Hospital
Staphylococcus aureus 61765	SA13	Turku University Hospital
Staphylococcus aureus 60881	SA14	Turku University Hospital
Staphylococcus aureus 60689	SA15	Turku University Hospital
