# Supplementary material for: On-Demand Isolation of Bacteriophages Against Drug-Resistant Bacteria for Personalized Phage Therapy
Source: Front Microbiol. 2015 Nov 13;6:1271. doi: 10.3389/fmicb.2015.01271 (PMC4643220; doi:10.3389/fmicb.2015.01271)
Supplement: Supplementary file 2 [file Table_2.PDF]

Table S2. Isolated phages.

Phage	Host	Plaque morphology	Titer	Titer after 1 month
AB1P1	*Acinetobacter baumannii* 57163	small, bright and some dim	4.2×10^{6}	3.0×10^{3}
AB1P2	*Acinetobacter baumannii* 57163	small, bright and some dim	2.0×10^{6}	7.2×10^{3}
AB2P1	*Acinetobacter baumannii* 59999	small, dim, rough edges, roundish	6.9×10^{7}	3.9×10^{7}
AB3P1	*Acinetobacter baumannii* 62060	medium-size, bright, big halo	4.5×10^{9}	9.1×10^{8}
AB3P2	*Acinetobacter baumannii* 62060	small, ,bright, round	1.8×10^{8}	3.5×10^{7}
AB6P1	*Acinetobacter baumannii* 18243	small and medium-size, dim, big halo	3.5×10^{8}	5.7×10^{8}
AB6P2	*Acinetobacter baumannii* 18243	small, dim, rough edges, roundish	1.1×10^{9}	1.5×10^{8}
AB8P1	*Acinetobacter baumanni* 18510	extremely small, slightly dim	1.5×10^{5}	2.6×10^{5}
AB8P2	*Acinetobacter baumannii* 18510	small, dim, rough edges, roundish	3.4×10^{5}	7.2×10^{4}
EC1P1	*Escherichia coli* 10AE5909	small, bright center surrounded by misty ring	6.0×10^{7}	5.6×10^{7}
EC2P1	*Escherichia coli* 12UM05186	big, bright	1.2×10^{10}	6.7×10^{9}
EC2P2	*Escherichia coli* 12UM05186	big, bright	7.6×10^{8}	1.3×10^{9}
EC3P1	*Escherichia coli* 10UU11258	medium-size, bright	1.7×10^{8}	8.4×10^{6}
EC3P2	*Escherichia coli* 10UU11258	medium-size, bright	4.6×10^{8}	1.3×10^{7}
EC4P1	*Escherichia coli* 11UT10019	small, bright	2.1×10^{7}	8.9×10^{6}
EC4P2	*Escherichia coli* 11UT10019	small, bright	1.6×10^{8}	2.6×10^{8}
EC5P1	*Escherichia coli* 11AN03027	small, bright	7.4×10^{6}	8.8×10^{5}
EC5P2	*Escherichia coli* 11AN03027	extremely small, bright	3.5×10^{8}	N/A
EC6P1	*Escherichia coli* 11UT12639	small, bright	1.6×10^{9}	2.9×10^{9}
EC6P2	*Escherichia coli* 11UT12639	small, bright	1.9×10^{9}	3.1×10^{9}
EC7P1	*Escherichia coli* 11UO03492	small, slightly dim	3.9×10^{9}	2.8×10^{9}
EC7P2	*Escherichia coli* 11UO03492	small, slightly dim	2.1×10^{10}	3.6×10^{10}
EC8P1	*Escherichia coli* 11UU07697	extremely small, slightly dim	2.4×10^{7}	7.3×10^{7}
EC8P2	*Escherichia coli* 11UU07697	extremely small, slightly dim	1.2×10^{7}	4.0×10^{7}
EC9P1	*Escherichia coli* 11UM05271	extremely small, bright	1.3×10^{9}	1.5×10^{9}
EC9P2	*Escherichia coli* 11UM05271	extremely small, bright	1.5×10^{10}	7.3×10^{9}
EC10P1	*Escherichia coli* 57262	small, bright	6.6×10^{10}	6.7×10^{10}
EC10P2	*Escherichia coli* 57262	small, bright	6.3×10^{10}	5.4×10^{10}
EC11P1	*Escherichia coli* 57294	big, bacterial growth in the middle	2.5×10^{9}	2.4×10^{8}
EC11P2	*Escherichia coli* 57294	small, some bright and some dim	1.6×10^{9}	4.0×10^{8}
EC12P1	*Escherichia coli* 57189	small, dim	1.2×10^{8}	8.5×10^{7}
EC12P2	*Escherichia coli* 57189	small, slightly dim	7.0×10^{7}	2.6×10^{7}
EC13P1	*Escherichia coli* 57253	small, dim	2.0×10^{8}	1.1×10^{8}
EC13P2	*Escherichia coli* 57253	small, dim	4.2×10^{8}	8.0×10^{7}
EC15P1	*Escherichia coli* 56895	small, bright, halo	9.0×10^{9}	9.6×10^{4}
EC15P2	*Escherichia coli* 56895	medium-size, dim, vague halo	2.3×10^{9}	4.0×10^{8}
EC16P1	*Escherichia coli* 57361	big and medium-size, bright, vague ring	3.7×10^{9}	6.3×10^{9}
EC16P2	*Escherichia coli* 57361	small/medium-size, bright	6.0×10^{8}	6.0×10^{8}
EF2P1	*Enterococcus faecalis* 58897	small, bright	3.8×10^{9}	1.7×10^{9}
EF2P2	*Enterococcus faecalis* 58897	small, bright	2.3×10^{9}	1.3×10^{9}
EF2P3	*Enterococcus faecalis* 58897	medium-size, brigth	8.8×10^{8}	7.3×10^{8}
EF4P1	*Enterococcus faecalis* ATCC 29212	big, bright, difficult to determine titer	3.5×10^{8}	N/A
EF5P1	*Enterococcus faecalis* ATCC 33186	big, bright, difficult to determine titer	7.0×10^{7}	N/A
EF6P1	*Enterococcus faecium* ATCC 9790	medium-size, bright, difficult to determine titer	8.0×10^{5}	N/A
EF9P2	*Enterococcus faecium* 61027	medium-size, round, dim, difficult to determine titer	4.0×10^{5}	N/A
KP1P1	*Klebsiella pneumoniae* DSM681	big, bacterial growth in the middle of plaque	6.8×10^{10}	1.7×10^{10}
KP1P2	*Klebsiella pneumoniae* DSM681	small, bright center surrounded by misty ring, some big	4.9×10^{8}	2.3×10^{7}
KP1P3	*Klebsiella pneumoniae* DSM681	medium-size, slightly turbid center	3.0×10^{10}	3.2×10^{9}
KP1P4	*Klebsiella pneumoniae* DSM681	medium-size, slightly turbid center	4.7×10^{10}	1.8×10^{10}
KP1P5	*Klebsiella pneumoniae* DSM681	big, turbid, bacterial growth in the middle of plaque	1.2×10^{11}	1.1×10^{10}
KP1P6	*Klebsiella pneumoniae* DSM681	variable size, some bright, some had bacterial growth in the middle	3.0×10^{10}	6.1×10^{9}
KP2P1	*Klebsiella pneumoniae* 10UO03898	medium-size, bright	1.5×10^{8}	2.5×10^{8}
KP2P2	*Klebsiella pneumoniae* 10UO03898	small, bright	2.6×10^{6}	2.1×10^{5}
KP3P2	*Klebsiella pneumoniae* 61705	medium-size, dim	1.0×10^{7}	1.5×10^{7}
KP4P1	*Klebsiella pneumoniae* 61784	small, bright, halo	1.4×10^{9}	4.4×10^{8}
KP5P1	*Klebsiella pneumoniae* 61837	small, bright	1.7×10^{9}	7.0×10^{8}
KP5P2	*Klebsiella pneumoniae* 61837	small, bright	5.0×10^{8}	2.9×10^{8}
KP6P1	*Klebsiella pneumoniae* 61794	small, slightly dim	4.0×10^{9}	1.8×10^{9}
KP6P2	*Klebsiella pneumoniae* 61794	small, dim	4.1×10^{8}	8.0×10^{7}
PA1P1	*Pseudomonas aeruginosa* 61841	small, bright, vague thin ring around	2.0×10^{11}	1.0×10^{11}
PA1P2	*Pseudomonas aeruginosa* 61841	small, bright, vague thin ring around	9.3×10^{10}	6.5×10^{10}
PA1P3	*Pseudomonas aeruginosa* 61841	small, bright, vague thin ring around	8.1×10^{10}	3.6×10^{10}
PA1P4	*Pseudomonas aeruginosa* 61841	small, bright, vague thin ring around	1.1×10^{11}	6.8×10^{10}
PA1P5	*Pseudomonas aeruginosa* 61841	small, bright, vague thin ring around	1.6×10^{11}	1.1×10^{11}
PA2P1	*Pseudomonas aeruginosa* 61823	small, dim	9.5×10^{8}	1.8×10^{11}
PA2P2	*Pseudomonas aeruginosa* 61823	small, round, turbid center surrounded by bright ring	5.0×10^{6}	5.0×10^{7}
PA3P1	*Pseudomonas aeruginosa* 61790	small/medium-size, dim	3.3×10^{10}	2.9×10^{8}
PA3P2	*Pseudomonas aeruginosa* 61790	small/medium-size, very dim, disfigured plaques	3.4×10^{9}	1.9×10^{9}
PA4P1	*Pseudomonas aeruginosa* 61432	small, dim	5.8×10^{6}	1.3×10^{7}
PA4P2	*Pseudomonas aeruginosa* 61432	round, bright	3.7×10^{9}	2.2×10^{9}
PA5P1	*Pseudomonas aeruginosa* 11AN03663	medium-size, bright	6.0×10^{9}	7.0×10^{9}
PA5P2	*Pseudomonas aeruginosa* 11AN03663	medium-size, bright, round, some small and dim	6.7×10^{8}	1.7×10^{6}
PA7P1	*Pseudomonas aeruginosa* 62314	small, dim, rough edges	2.1×10^{10}	3.9×10^{10}
PA7P2	*Pseudomonas aeruginosa* 62314	medium-size, slightly dim	1.5×10^{11}	5.5×10^{10}
PA8P1	*Pseudomonas aeruginosa* 62263	small, halo	1.4×10^{11}	1.3×10^{11}
PA8P2	*Pseudomonas aeruginosa* 62263	small, bright	6.1×10^{8}	2.9×10^{6}
PA9P1	*Pseudomonas aeruginosa* 62224	small, bright, halo	1.4×10^{11}	1.9×10^{11}
PA9P2	*Pseudomonas aeruginosa* 62224	big, bright	1.2×10^{9}	3.4×10^{7}
PA10P1	*Pseudomonas aeruginosa* 62206	big, dim	7.0×10^{9}	6.0×10^{9}
PA10P2	*Pseudomonas aeruginosa* 62206	small, bright, halo	1.0×10^{11}	1.1×10^{11}
PA10P3	*Pseudomonas aeruginosa* 62206	small, dim, vague halo	6.7×10^{10}	6.8×10^{9}
PA11P1	*Pseudomonas aeruginosa* 62180	small, dim, rough edges	2.0×10^{11}	1.4×10^{11}
PA11P2	*Pseudomonas aeruginosa* 62180	small, round, dim	8.0×10^{11}	5.4×10^{11}
PA12P1	*Pseudomonas aeruginosa* 62181	medium-size, slightly dim, rough edges	1.7×10^{8}	2.5×10^{8}
PA12P2	*Pseudomonas aeruginosa* 62181	small/medium-size, slightly dim, round	1.4×10^{11}	1.2×10^{11}
PA13P1	*Pseudomonas aeruginosa* 62172	medium-size, bright	1.2×10^{11}	1.1×10^{11}
PA13P2	*Pseudomonas aeruginosa* 62172	small, dim, some had halo	2.7×10^{11}	4.2×10^{11}
PA14P1	*Pseudomonas aeruginosa* 62109	extremely small, slightly dim	2.6×10^{6}	2.1×10^{4}
PA14P2	*Pseudomonas aeruginosa* 62109	big, slightly dim, round	4.1×10^{9}	4.2×10^{9}
PA16P1	*Pseudomonas aeruginosa* 62069	extremely small, dim, disfigured plaques, difficult to determine titer	1.4×10^{8}	6.7×10^{7}
PA16P2	*Pseudomonas aeruginosa* 62069	small, round (slightly rough edges), dim	9.5×10^{7}	6.6×10^{7}
PA17P1	*Pseudomonas aeruginosa* 61932	small/medium-size, slightly dim,	3.0×10^{9}	3.9×10^{9}
PA17P2	*Pseudomonas aeruginosa* 61932	small/medium-size, slightly dim, round, halo	2.4×10^{9}	1.9×10^{9}
SA10P1	*Staphylococcus aureus* 10AE05905	small, slightly dim	7.4×10^{8}	2.3×10^{9}
SA10P2	*Staphylococcus aureus* 10AE05905	small, slightly dim	1.5×10^{9}	7.0×10^{9}
SA10P3	*Staphylococcus aureus* 10AE05905	small, slightly dim, roundish	4.0×10^{8}	1.0×10^{8}
SA10P4	*Staphylococcus aureus* 10AE05905	small, dim, round	5.0×10^{7}	3.3×10^{7}
SA14P1	*Staphylococcus aureus* 60881	small, slightly dim	4.7×10^{7}	1.0×10^{6}
S1P1	*Salmonella* , quality control strain 18048	small, dim, round	1.2×10^{10}	2.1×10^{10}
SB1P1	*Salmonella* , Group B, ESBL-strain 12FB01687	small and medium-size, dim, roundish	3.5×10^{8}	1.8×10^{8}
SC1P1	*Salmonella* , Group C, 13FB10784	extremely small, bright, round	1.3×10^{10}	3.0×10^{6}
SE1P1	*Salmonella enteritis* (36.)	small, dim	1.1×10^{8}	9.2×10^{7}
SE1P2	*Salmonella enteritis* (36.)	in high density: small, dim, round / in lower density: big, dim halo, bright center, round	1.3×10^{7}	1.0×10^{8}
SE3P1	*Salmonella enteritidis* FB11214	big, turbid center, bright halo, round	7.8×10^{7}	5.0×10^{7}
SK1P1	*Salmonella Kreber* (37.)	extremely small, bright	1.9×10^{7}	9.8×10^{6}
SK1P2	*Salmonella Kreber* (37.)	extremely small, slightly dim, round	2.9×10^{6}	1.6×10^{6}
SS1P1	*Salmonella stanley* FB3820	small, extremely dim, roundish	N/A	N/A
ST1P1	*Salmonella typhimurium* FB7595	big, turbid center, bright halo, round	1.2×10^{10}	2.4×10^{10}
